# Association of Platelet Indices With Vascular Complications in Type 2 Diabetes Mellitus: A Hospital-Based Cross-Sectional Study

**DOI:** 10.7759/cureus.117826

**Published:** 2026-10-12

**Authors:** Pratibha Malviya, Rajni Choudhary, Sonal Gupta, Rita Singh Saxena, Maneesh Sulya

**Affiliations:** 1 Department of Pathology, Gandhi Medical College and Hamidia Hospital, Bhopal, IND; 2 Department of Medicine, Gandhi Medical College and Hamidia Hospital, Bhopal, IND

**Keywords:** cross-sectional study, mean platelet volume(mpv), platelet activation, platelet indices, platelet-large cell ratio, type 2 diabetes mellitus, vascular complications

## Abstract

Background: Platelet indices are inexpensive markers of platelet size and heterogeneity that may reflect the prothrombotic state of type 2 diabetes mellitus (T2DM). This study compares routinely reported platelet indices between adults with T2DM with and without documented prevalent microvascular or macrovascular complications and evaluates the adjusted associations of mean platelet volume (MPV) and platelet large cell ratio (P-LCR) with complication status.

Methods: This hospital-based cross-sectional study included 106 unique participants with T2DM, conducted at the Gandhi Medical College and associated Hamidia Hospital, Bhopal, India, from May 2024 to October 2025. Any complication was reconstructed as the logical OR of four component indicators (neuropathy, nephropathy, retinopathy, and macrovascular disease) after a master-chart audit. Group comparisons used two-sided Mann-Whitney U tests with rank-biserial effects and Holm adjustment across four platelet indices. Separate logistic-regression models for MPV and P-LCR adjusted for age, sex, and HbA1c category; coefficients were evaluated with two-sided Wald z tests.

Results: Thirty-three of 106 participants (31.1%) had at least one component-defined complication. Both MPV and P-LCR were higher with complications (MPV: 11.28±1.95 versus 10.53±1.68 fL, U=1506.0, unadjusted p=0.040, Holm p=0.120; P-LCR: 34.10±12.45% versus 27.38±8.56%, U=1599.0, unadjusted p=0.007, Holm p=0.029). Adjusted P-LCR remained associated with complications (adjusted odds ratio (aOR) 1.44 per five-percentage-point increase, 95% CI 1.13-1.85; Wald z=2.92, p=0.003), whereas adjusted MPV was borderline (aOR 1.27 per 1 fL, 95% CI 0.99-1.63; Wald z=1.90, p=0.058).

Conclusion: The P-LCR showed the most consistent association with prevalent vascular complications, while the MPV finding was less robust after multiplicity correction and covariate adjustment. These cross-sectional results support further prospective validation but do not establish causality, prediction, diagnostic superiority, or incremental value beyond clinical assessment and established complication-screening methods.

## Introduction

Type 2 diabetes mellitus (T2DM) is a major global health problem, and much of its morbidity is attributable to microvascular and macrovascular complications. Chronic hyperglycemia, oxidative stress, endothelial dysfunction, inflammation, and abnormalities of hemostasis create a biologic environment favorable to vascular injury and thrombosis [1,2].

Platelets participate in this process through enhanced adhesiveness, activation, aggregation, and altered intracellular signaling. Larger platelets are generally more metabolically and enzymatically active and contain more prothrombotic mediators than smaller platelets. Consequently, changes in platelet size and turnover may accompany the vascular manifestations of T2DM [2,3]. Mean platelet volume (MPV), platelet distribution width (PDW), platelet count, and platelet large cell ratio (P-LCR) are routinely generated by automated hematology analyzers. Prior hospital-based studies have reported inconsistent associations between these indices and diabetic complications, partly because populations, analyzers, outcome definitions, pre-analytical timing, and covariate adjustment differ [3-10].

The objectives of this study were to determine whether routinely reported platelet indices, i.e., MPV, PDW, platelet count, and P-LCR, differed between participants with T2DM who had versus did not have documented prevalent microvascular or macrovascular complications, and to estimate the associations of MPV and P-LCR with complication status after adjustment for age, sex, and HbA1c category. These indices were evaluated as supplementary laboratory markers and not as substitutes for clinical history, physical examination, or established complication-screening methods.

## Materials and methods

Study design, setting, and reporting framework

This hospital-based cross-sectional study was conducted in the Department of Pathology, Gandhi Medical College and Hamidia Hospital (Bhopal, MP, IND), over 18 months from May 2024 to October 2025. Patients were recruited from the medicine outpatient department and medicine wards. Reporting was organized according to the Strengthening the Reporting of Observational Studies in Epidemiology (STROBE) statement [11]. This study was approved by the Institutional Ethics Committee (IEC) (approval no. 193/IEC/2024). Written informed consent was obtained from all participants before enrollment and sample collection.

Participants and study size

Patients diagnosed with diabetes mellitus who attended or were admitted to the participating medicine services and provided written informed consent were eligible. Patients with known bleeding disorders or other conditions expected to substantially alter platelet indices were excluded. This study was conducted per the IEC-approved MD (Pathology) thesis protocol titled 'Study of Platelet Indices in Patients of Diabetes Mellitus'. The protocol calculated the sample size using $n = \frac{4pq}{d^2}$, with an expected diabetes prevalence (p) of 11.6%, q=88.4%, and an allowable error (d) of 6.5%. This yielded a minimum sample size of 97, which was rounded to a recruitment target of 100. The final analysis included 106 unique participants. This prevalence-based calculation established the recruitment target for the underlying thesis and was not an association-specific power calculation. Given the 33 participants with the composite outcome, the adjusted regression analyses were considered exploratory and hypothesis-generating.

Variables and outcome definitions

The primary outcome was the presence of at least one predefined vascular complication. Diabetic nephropathy was defined as albuminuria greater than 300 mg/24 hours or an albumin-to-creatinine ratio greater than 300 mg/g. Diabetic neuropathy was identified by clinical neurological examination and nerve-conduction studies. Diabetic retinopathy was identified by fundoscopic examination. Macrovascular complications included documented coronary artery disease, stroke, or peripheral vascular disease.

The exposures were MPV (fL), PDW (fL), platelet count (×10³/µL), and P-LCR (%). Prespecified adjustment variables were age in years, sex, and HbA1c category (≤6.5% or >6.5%). The dataset contained two HbA1c results reported as >14%; they were retained in the >6.5% category but excluded from analyses requiring exact numerical HbA1c. Other potentially relevant covariates, including diabetes duration, hypertension, kidney function, body mass index, smoking status, family history of vascular disease, and medication use, including antiplatelet therapy, were not comprehensively and uniformly recorded in the available dataset. They were therefore not included in the adjusted models.

Specimen collection and laboratory measurements

Venous blood was collected under aseptic precautions. Ethylenediaminetetraacetic acid (EDTA) specimens used for complete blood count and platelet-index measurements were processed within one to three hours of collection to reduce time-dependent platelet swelling. Platelet count, MPV, PDW, and P-LCR were measured using the Sysmex KX-21 (Sysmex Corp., Kobe, JPN), a three-part automated hematology analyzer. The laboratory or manufacturer reference intervals were 150-450 × 10³/μL for platelet count, 8.0-12.0 fL for MPV, 9.0-17.0 fL for PDW, and 15.0-35.0% for P-LCR.

Internal quality control was performed daily using manufacturer-provided tri-level commercial controls. The laboratory participated in an external quality-assurance program for hematology during the study period; the External Quality Assessment Scheme (EQAS) was provided by Bio-Rad Laboratories. The HbA1c was measured using high-performance liquid chromatography, and plasma glucose was measured using the hexokinase method. Fasting status was defined as an overnight fast of eight to 12 hours and confirmed by patient verbal confirmation prior to phlebotomy. All participants had complete age, sex, HbA1c category, and platelet-index data. Standardized processing time and predefined clinical criteria reduced measurement variability, but selection bias and residual confounding remained possible.

Statistical analysis

Continuous variables were summarized as mean±SD and median with IQR; categorical variables were summarized as number and percentage. Two-group comparisons used the two-sided Mann-Whitney U test because several laboratory variables were non-normally distributed; the U statistic is reported with each p-value. Rank-biserial correlation quantified unadjusted effect size, with positive values indicating higher measurements in the complication group. Binary categorical variables were assessed using two-sided Fisher exact tests. Odds ratios accompany Fisher's exact p-values as effect estimates because Fisher's exact test does not produce a separate t, χ², or F statistic.

The four platelet-index p-values were adjusted using the Holm procedure to address multiplicity. MPV and P-LCR were strongly correlated (Spearman ρ=0.750; p<0.001) and were therefore entered into separate exploratory logistic-regression models. Each model included one platelet index, age, sex, and HbA1c category. Model coefficients were evaluated with two-sided Wald z tests and are reported with the Wald z statistic, adjusted odds ratio (aOR), 95% confidence interval (CI), and p-value. MPV was scaled per 1 fL, and P-LCR per five percentage points. The models included 33 outcome events and four predictor degrees of freedom, so estimates were interpreted cautiously. Analyses were independently reproduced with Python version 3.12 (Python Software Foundation, Wilmington, DE, USA), pandas version 3.0.1, and NumPy version 2.3.5. Two-sided p <0.05 was the nominal threshold.

Data availability

The participant-level dataset is not publicly available because the original consent and ethics approval did not provide for unrestricted public release. De-identified data may be made available by the corresponding author upon reasonable request and subject to institutional and ethics approval. 

## Results

Study population

The analysis included 106 unique participants. The four component variables identified 33 participants (31.1%) with any vascular complication and 73 (68.9%) without a documented complication. There were 34 component events because one participant had both nephropathy and macrovascular disease. The corrected parallel participant flow is shown in Figure 1. Both models used n=106 and 33 events. The MPV-model area under the receiver operating characteristic (AUROC) curve was 0.660; the P-LCR-model value was 0.705. These values describe apparent fit and were not internally or externally validated.

**Figure 1:**
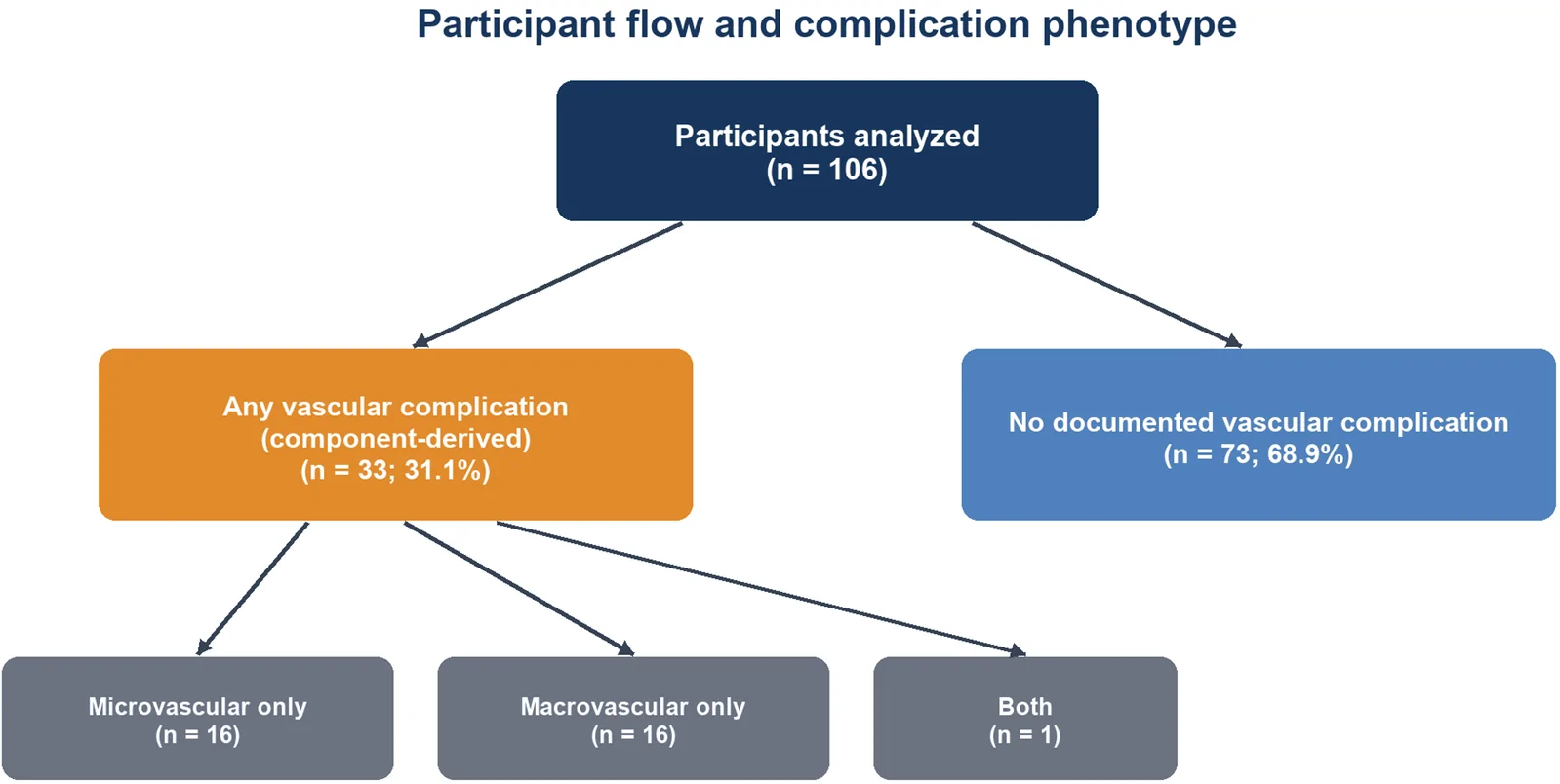
Participant flow and mutually exclusive complication phenotypes based on the component-derived outcome

Macrovascular disease was the most frequent component (17 participants), followed by nephropathy (10), neuropathy (four), and retinopathy (three). Figure 2 displays these non-exclusive category counts. Table 1 summarizes demographic and glycemic characteristics by corrected complication status. None of the tested baseline variables differed significantly between groups.

**Figure 2:**
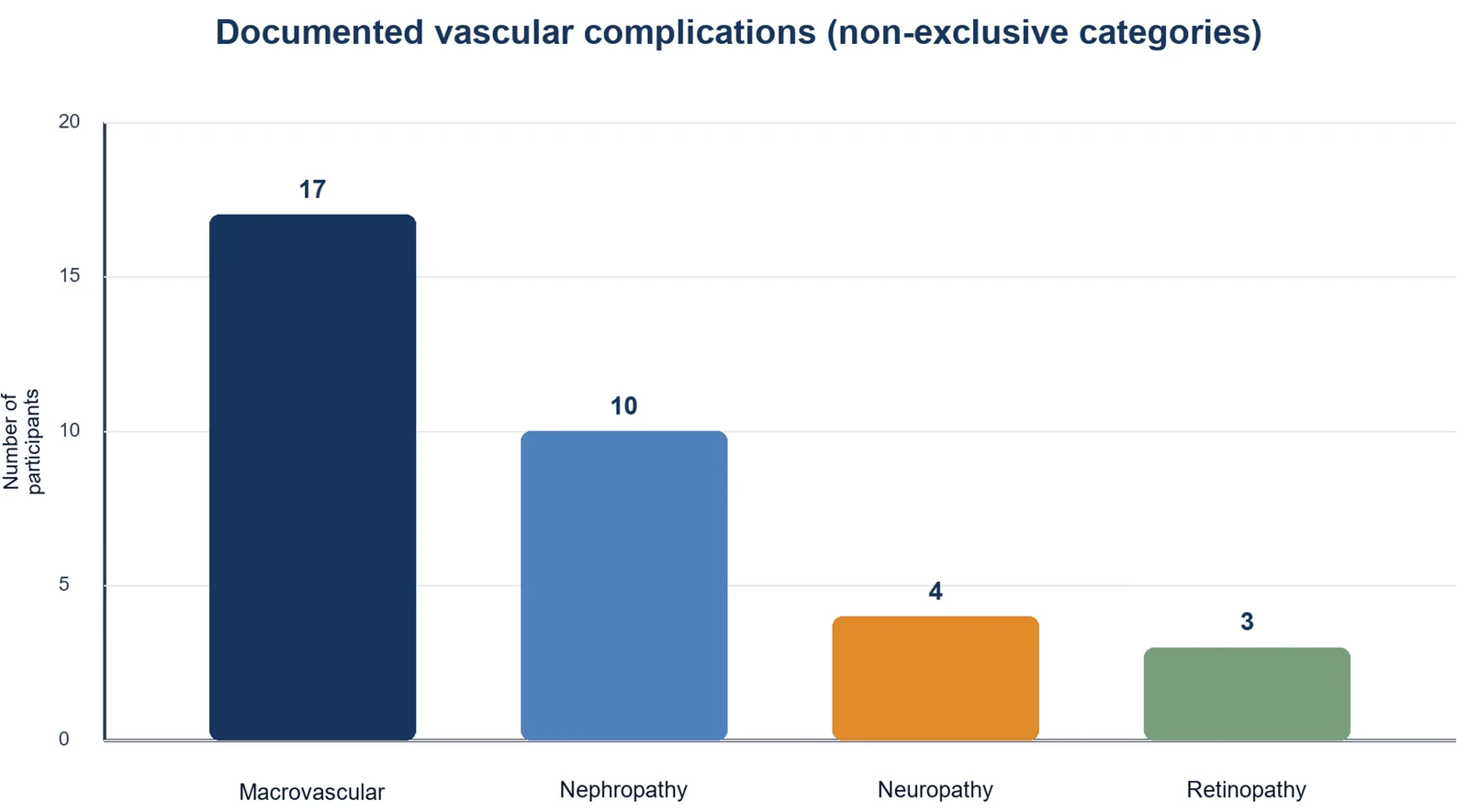
Distribution of documented vascular complications Categories are non-exclusive because one participant had both nephropathy and macrovascular disease.

**Table 1:** Demographic and glycemic characteristics by component-derived complication status *Exact numerical HbA1c was available for 31 participants with complications and all 73 participants without complications. Continuous-variable p-values and U statistics are from two-sided Mann-Whitney U tests. Categorical-variable p-values are from two-sided Fisher exact tests; the accompanying OR is an effect estimate because Fisher's exact test has no separate t, χ², or F statistic. The age median row is descriptive and does not represent an additional test.

Characteristic	Complication present (n=33)	Complication absent (n=73)	p-value	Statistic or effect
Age, years, mean±SD	48.61±13.47	49.32±13.98	0.978	U=1209.0
Age, median (IQR)	52.0 (37.0-60.0)	46.0 (39.0-58.0)	—	Descriptive only
Male sex, n (%)	13 (39.4)	41 (56.2)	0.143	OR=0.51
HbA1c >6.5%, n (%)	24 (72.7)	47 (64.4)	0.505	OR=1.48
Exact HbA1c, %, mean±SD*	8.80±3.23	8.45±2.95	0.644	U=1197.0
Fasting glucose, mg/dL, mean±SD	163.15±101.60	144.01±71.83	0.419	U=1323.5
Postprandial glucose, mg/dL, mean±SD	213.70±113.19	208.81±85.71	0.764	U=1160.0

Platelet indices

MPV and P-LCR were higher in participants with complications (MPV, U=1506.0, p=0.040; P-LCR, U=1599.0, p=0.007), while PDW (U=1134.5, p=0.633) and platelet count (U=1121.5, p=0.573) were similar. After Holm adjustment for the four platelet comparisons, only P-LCR remained statistically significant. Table 2 presents means, medians, Mann-Whitney U statistics, effect sizes, nominal p-values, and multiplicity-adjusted p-values. The distributions of MPV and P-LCR, including individual observations, are shown in Figure 3.

**Table 2:** Platelet indices by component-derived complication status The U and unadjusted p-values are from two-sided Mann-Whitney U tests. Holm p-values adjust the four platelet-index comparisons. Rank-biserial r is the unadjusted effect size. P-LCR: Platelet large cell ratio; MPV: Mean platelet volume; PDW: Platelet distribution width

Characteristic	Complication present (n=33)	Complication absent (n=73)	p-value	Statistic or effect
Age, years, mean±SD	48.61±13.47	49.32±13.98	0.978	U=1209.0
Age, median (IQR)	52.0 (37.0-60.0)	46.0 (39.0-58.0)	—	Descriptive only
Male sex, n (%)	13 (39.4)	41 (56.2)	0.143	OR=0.51
HbA1c >6.5%, n (%)	24 (72.7)	47 (64.4)	0.505	OR=1.48
Exact HbA1c, %, mean±SD*	8.80±3.23	8.45±2.95	0.644	U=1197.0
Fasting glucose, mg/dL, mean±SD	163.15±101.60	144.01±71.83	0.419	U=1323.5
Postprandial glucose, mg/dL, mean±SD	213.70±113.19	208.81±85.71	0.764	U=1160.0

**Figure 3:**
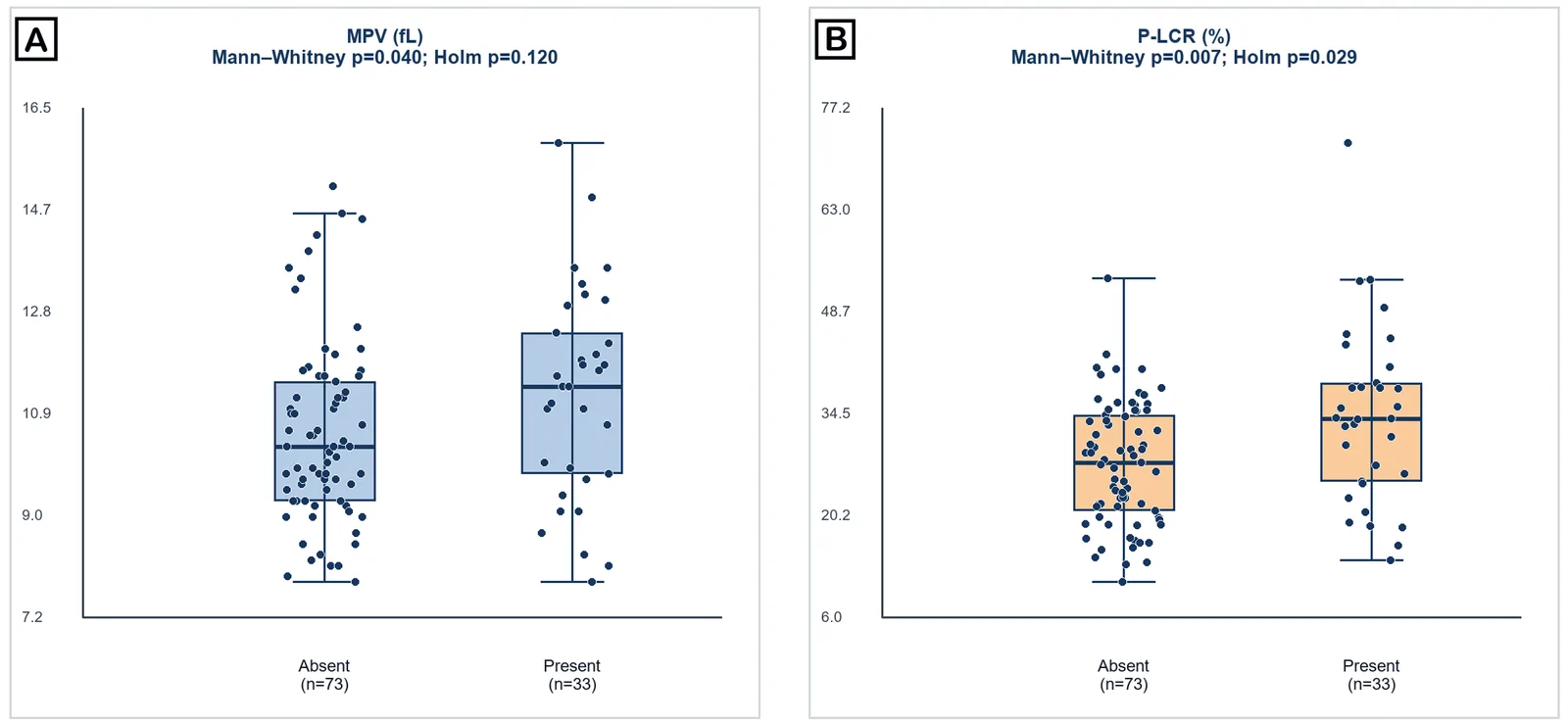
MPV and P-LCR distributions by corrected complication status Boxes show the IQR, central lines show medians, whiskers extend to 1.5×IQR, and points represent individual participants. MPV: Mean platelet volume; P-LCR: Platelet large cell ratio

Adjusted analysis

In separate adjusted logistic-regression models, P-LCR remained associated with complication status (aOR 1.44 per five-percentage-point increase, 95% CI 1.13-1.85; Wald z=2.92, p=0.003). The MPV association was borderline (aOR 1.27 per 1 fL, 95% CI 0.99-1.63; Wald z=1.90, p=0.058). Table 3 reports the Wald z statistic, aOR, 95% CI, and p-value for every model coefficient.

**Table 3:** Logistic-regression models for component-derived vascular complication status aOR: Adjusted odds ratio; MPV: Mean platelet volume; P-LCR: Platelet large cell ratio

Model	Predictor	aOR	95% CI	p-value	Wald z
MPV model	MPV, per 1 fL	1.27	0.99-1.63	0.058	1.90
MPV model	Age, per year	0.99	0.96-1.02	0.615	-0.50
MPV model	Male vs female	0.50	0.21-1.19	0.116	-1.57
MPV model	HbA1c >6.5% vs ≤6.5%	1.06	0.40-2.84	0.904	0.12
P-LCR model	P-LCR, per 5 percentage points	1.44	1.13-1.85	0.003	2.92
P-LCR model	Age, per year	0.98	0.95-1.02	0.372	-0.89
P-LCR model	Male vs female	0.47	0.19-1.17	0.103	-1.63
P-LCR model	HbA1c >6.5% vs ≤6.5%	1.09	0.40-2.99	0.861	0.18

Both models used n=106 and 33 events. Wald z and p-values are from two-sided Wald tests in separate binary logistic-regression models. The MPV-model area under the receiver operating characteristic curve was 0.660; the P-LCR-model value was 0.705. These values describe apparent fit and were not internally or externally validated. Figure 4 summarizes the adjusted index effects from the 106-participant analysis.

**Figure 4:**
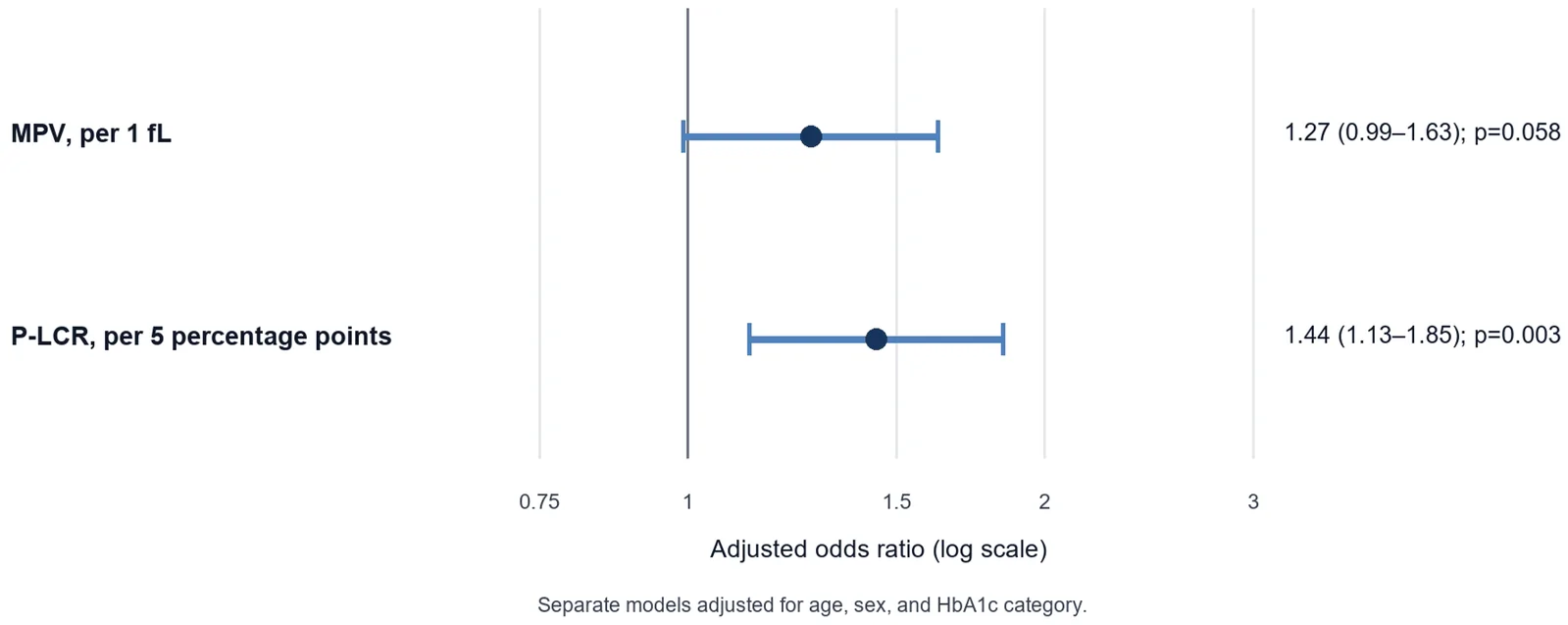
The aORs for MPV and P-LCR from separate logistic-regression models Horizontal bars show 95% confidence intervals. Separate models were adjusted for age, sex, and HbA1c category. aORs: Adjusted odds ratios; MPV: Mean platelet volume; P-LCR: Platelet large cell ratio

## Discussion

The main finding was a consistent association between P-LCR and prevalent vascular complications. The P-LCR was higher in the complication group, remained significant after adjustment for four platelet-index comparisons, and retained an adjusted association after accounting for age, sex, and HbA1c category. The MPV difference was nominally significant but did not survive Holm correction, and its adjusted CI included the null. The PDW and platelet count were not associated with the composite outcome. Because the study was cross-sectional and several clinically relevant covariates were unavailable, these findings represent observed associations under limited adjustment. They should not be interpreted as evidence of causality, independent predictive value, or superiority to standard clinical assessment.

The P-LCR result is biologically plausible because P-LCR quantifies the proportion of large circulating platelets, which tend to be more reactive. However, MPV and P-LCR were strongly correlated, and the study cannot establish that either index has an independent mechanistic role. The indices were therefore modeled separately rather than interpreted as simultaneous independent predictors.

Comparison with previous studies

Table 4 places the current findings beside selected, closely related studies. Differences in outcome definition and adjustment are important when comparing effect estimates.

**Table 4:** Comparison with selected studies of platelet indices and diabetic complications T2DM: Type 2 diabetes mellitus; MPV: Mean platelet volume; PDW: Platelet distribution width; P-LCR: Platelet large cell ratio; aOR: Adjusted odds ratio

Study	Design/sample	Main finding	Relation to present study
Shilpi and Potekar, 2018 [3]	Hospital study; 280 T2DM and 280 controls	MPV, PDW, and P-LCR were higher in T2DM than controls, but complication subgroup differences were not significant	Shows that diabetic-status differences do not necessarily translate into robust complication discrimination
Buch et al., 2017 [5]	Case-control; 300 T2DM and 200 controls	MPV and PDW increased with complications; P-LCR was not significant	Supports MPV but contrasts with the stronger P-LCR signal in the present study
Walinjkar et al., 2019 [4]	Hospital study; 125 T2DM plus matched controls	MPV, PDW, and P-LCR were higher with microvascular complications	Broadly supports platelet-size indices, although the present PDW result was null
Taderegew et al., 2021 [6]	Cross-sectional; 352 T2DM; 117 with microvascular complications	Adjusted associations for MPV, PDW, and P-LCR; P-LCR aOR 1.07 per 1 percentage point	Most directly supports the present adjusted P-LCR association; the current per-point equivalent is approximately 1.08
Ji et al., 2019 [12]	Systematic review and meta-analysis; 14 studies; 1,252 with diabetic retinopathy and 1,359 T2DM without retinopathy	MPV was higher in diabetic retinopathy than in T2DM without retinopathy (standardized mean difference 0.36, 95% CI 0.19-0.53)	Supports an MPV-retinopathy association, but the present study had only three retinopathy cases and used a combined vascular outcome
Sengupta et al., 2024 [13]	Hospital cross-sectional study; 203 patients with T2DM and nephropathy across stages 1-5	MPV, PDW, plateletcrit (PCT), and P-LCR differed across nephropathy stages (Kruskal-Wallis p=0.027, 0.009, 0.001, and 0.007, respectively)	Directionally supports P-LCR; differences in renal-stage outcome, analyzer, and case mix may explain the present null PDW result
Present study	Cross-sectional; 106 participants; 33 with any microvascular or macrovascular complication	P-LCR robust after multiplicity and limited adjustment; MPV weaker; PDW and count null	Extends the comparison to a combined vascular outcome but has fewer events and less confounder information

The closest adjusted comparison is Taderegew et al., who studied 352 adults with T2DM, including 117 with microvascular complications, and reported an adjusted P-LCR odds ratio of 1.07 per one-percentage-point increase after adjustment for age, sex, diabetes duration, anthropometry, blood pressure, hypertension, and fasting glucose [6]. Rescaling the present estimate of 1.44 per five percentage points gives an approximate per-point aOR of 1.08, which is similar in magnitude. Direct equivalence remains limited because the earlier study examined microvascular complications only, whereas the present outcome combined microvascular and macrovascular disease and used a smaller adjustment set. Conversely, Buch et al. found significant MPV and PDW differences but no significant P-LCR difference [5], while Shilpi and Potekar found higher MPV, PDW, and P-LCR in complication subgroups without statistically significant between-subgroup differences [3]. These differences show that the relative performance of platelet indices varies across populations and study methods.

The meta-analysis by Ji et al. pooled 14 studies and found higher MPV in diabetic retinopathy than in T2DM without retinopathy (standardized mean difference 0.36, 95% CI 0.19-0.53) [12]. That complication-specific result is compatible with a platelet-size signal, but the present study included only three participants with retinopathy and therefore cannot test a retinopathy-specific association. Sengupta et al. evaluated 203 patients with diabetic nephropathy and found that MPV, PDW, plateletcrit (PCT), and P-LCR differed across nephropathy stages, with Kruskal-Wallis p-values of 0.027, 0.009, 0.001, and 0.007, respectively [13]. Their severity-based renal outcome differs from the present binary composite, which may partly explain why PDW was null here. Together with the conflicting findings from Buch et al. and Shilpi and Potekar, the literature indicates that outcome definition, complication mix, analyzer platform, specimen-processing interval, and covariate adjustment can materially affect observed associations. Accordingly, the current P-LCR result should be viewed as supportive rather than as evidence for a universal cutoff.

Two recent reviews provide additional context. Jena et al. described higher MPV, PDW, P-LCR, and plateletcrit as recurring findings in diabetes, particularly with poor glycemic control or vascular complications, but emphasized that longitudinal studies are needed before these indices can be used for risk prediction [14]. Mirghani likewise concluded that platelet indices are inexpensive and readily available adjuncts but are nonspecific and can be influenced by age, renal impairment, medication use, hemoglobin, and diabetes duration [15]. These cautions are consistent with the present study's interpretation of P-LCR as a supplementary marker rather than a standalone diagnostic or prognostic test.

Clinical interpretation

The P-LCR is routinely available from many complete blood count analyzers and may provide supplementary information about platelet size distribution. In this study, it was associated with documented prevalent vascular complications; however, diagnostic thresholds were not evaluated, the apparent model performance was not internally or externally validated, and the study did not assess whether P-LCR added information beyond clinical history, physical examination, conventional risk factors, or established screening procedures. The P-LCR should therefore be interpreted only as a potential supplementary laboratory marker and should not replace careful clinical assessment or established screening for retinopathy, nephropathy, neuropathy, coronary disease, cerebrovascular disease, or peripheral vascular disease. The present findings do not support initiating or modifying antiplatelet therapy, glycemic targets, referral decisions, or complication-screening intervals on the basis of P-LCR or any other platelet index.

Strengths and limitations

Strengths include access to individual-level data, a documented specimen-processing interval, confirmation that all 106 entries represented unique participants, an explicit audit of outcome counts, effect-size reporting, multiplicity adjustment, and parsimonious adjusted models. Reconstructing the composite outcome from its component indicators made the complication count internally consistent. An additional practical strength is that the platelet indices evaluated are routinely generated by many automated hematology analyzers and may therefore be available without additional blood collection or a specialized assay, although their availability and comparability depend on the analyzer platform and pre-analytical conditions.

Several limitations are substantial. This study was a single-center, hospital-based cross-sectional study, so temporality, causality, and prediction of future complications cannot be inferred. The small numbers of neuropathy and retinopathy events precluded reliable complication-specific modeling. Recruitment was limited to patients attending or admitted to the medicine services, and the exclusion criterion for conditions expected to alter platelet indices was broad. The study did not construct a clinical-history-based comparison model or evaluate whether P-LCR provided incremental information beyond a careful clinical history, physical examination, conventional vascular risk factors, or established complication-screening methods. The findings should therefore not be interpreted as evidence that platelet indices identify vascular complications more effectively than standard clinical assessment.

Adjustment was limited to age, sex, and HbA1c category because diabetes duration, hypertension, kidney function, body mass index, smoking status, family history of vascular disease, and medication use, including antiplatelet therapy, were not comprehensively and uniformly available. Residual confounding is therefore likely. The sample-size calculation was prevalence-based rather than designed specifically to detect platelet-index associations, and only 33 participants had the composite outcome. Consequently, the adjusted regression models should be interpreted as exploratory.

Future research

Future studies should use an adequately powered, prospective, multicenter longitudinal design to determine whether platelet indices are associated with incident rather than prevalent vascular complications. These studies should use standardized analyzer platforms, quality-control procedures, specimen-processing intervals, and outcome definitions. They should also collect comprehensive clinical history and conventional risk-factor data, including diabetes duration, smoking, family history, blood pressure, body mass index, kidney function, medication use, and antiplatelet therapy. A conventional clinical model should first be developed using established clinical variables, after which P-LCR or other platelet indices should be added to determine whether they provide meaningful incremental value. Model discrimination, calibration, and clinical usefulness should be assessed through prespecified internal validation and subsequent external validation before any platelet index is considered for treatment or screening decisions.

## Conclusions

The P-LCR showed a consistent cross-sectional association with prevalent vascular complications in this hospital-based T2DM dataset after multiplicity correction and limited covariate adjustment. The MPV showed a weaker association that was not robust to multiplicity correction and was borderline after adjustment; PDW and platelet count were not associated. These indices may be considered supplementary laboratory markers, but they are not validated standalone diagnostic or prognostic tests. Larger multicenter longitudinal studies should use standardized analyzers, collect comprehensive clinical history and conventional risk-factor data, and determine whether P-LCR provides incremental information beyond clinical assessment and established complication-screening methods before it is considered for clinical decision-making.

## References

[1] Sun H, Saeedi P, Karuranga S (2022). IDF Diabetes Atlas: global, regional and country-level diabetes prevalence estimates for 2021 and projections for 2045. Diabetes Res Clin Pract.

[2] Stratmann B, Tschoepe D (2005). Pathobiology and cell interactions of platelets in diabetes. Diab Vasc Dis Res.

[3] Shilpi K, Potekar RM (2018). A study of platelet indices in type 2 diabetes mellitus patients. Indian J Hematol Blood Transfus.

[4] Walinjkar RS, Khadse S, Kumar S, Bawankule S, Acharya S (2019). Platelet indices as a predictor of microvascular complications in type 2 diabetes. Indian J Endocrinol Metab.

[5] Buch A, Kaur S, Nair R, Jain A (2017). Platelet volume indices as predictive biomarkers for diabetic complications in type 2 diabetic patients. J Lab Physicians.

[6] Taderegew MM, Woldeamanuel GG, Emeria MS, Tilahun M, Yitbarek GY, Zegeye B (2021). Platelet indices and its association with microvascular complications among type 2 diabetes mellitus patients in northeast Ethiopia: a cross-sectional study. Diabetes Metab Syndr Obes.

[7] Ahluwalia RS, Jagtap SV, Borade DS, Khawale R, Billawaria S (2024). Association of platelet indices with diabetic complications: a cross-sectional study. J Clin Diagn Res.

[8] Bhattacharjee P, Datta A, Debbarma RK, Das SK (2016). Platelet indices in diabetics and influence of glycemic control — a hospital-based study in North-East India. Int J Med Res Rev.

[9] Kshirsagar R, Deoke S, Akhtar S (2019). Platelet indices in type 2 diabetes mellitus and their association with microvascular complications. Panacea J Med Sci.

[10] Iqbal S, Arbab KN, Khattak MT, Ali A, Banori A, Khan MI (2022). A study of platelet indices in type 2 diabetes mellitus patients: a cross-sectional study design. Pak J Med Health Sci.

[11] von Elm E, Altman DG, Egger M, Pocock SJ, Gøtzsche PC, Vandenbroucke JP (2008). The Strengthening the Reporting of Observational Studies in Epidemiology (STROBE) statement: guidelines for reporting observational studies. J Clin Epidemiol.

[12] Ji S, Zhang J, Fan X (2019). The relationship between mean platelet volume and diabetic retinopathy: a systematic review and meta-analysis. Diabetol Metab Syndr.

[13] Sengupta P, Priyadarshini A, Behera PK, Tripathy KP (2024). Exploring platelet indices as predictors of nephropathy severity in type 2 diabetes mellitus: a hospital-based cross-sectional analysis. Cureus.

[14] Jena S, Raizada N, Kalra S, Bhattacharya S (2025). Platelet indices as predictors of glycaemic status and complications in diabetes mellitus. Apollo Med.

[15] Mirghani HO (2025). Platelets indices clinical implications in diabetes mellitus: a broader insight. World J Diabetes.

